# Drinking water nitrate and risk of pregnancy loss: a nationwide cohort study

**DOI:** 10.1186/s12940-022-00897-1

**Published:** 2022-09-16

**Authors:** Ninna Hinchely Ebdrup, Jörg Schullehner, Ulla Breth Knudsen, Zeyan Liew, Anne Marie Ladehoff Thomsen, Julie Lyngsø, Bjørn Bay, Linn Håkonsen Arendt, Pernille Jul Clemmensen, Torben Sigsgaard, Birgitte Hansen, Cecilia Høst Ramlau-Hansen

**Affiliations:** 1 Department of Obstetrics and Gynecology, Horsens Fertility Clinic, Horsens, Denmark; 2 Department of Public Health, Aarhus University, Aarhus, Denmark; 3 Department of Clinical Medicine, Aarhus University, Aarhus, Denmark; 4 Geological Survey of Denmark and Greenland, Aarhus, Denmark; 5 Department of Environmental Health Sciences, Yale School of Public Health, New Haven, CT USA; 6 Yale Center for Perinatal, Pediatric, and Environmental Epidemiology, Yale School of Public Health, New Haven, CT USA; 7 DEFACTUM - Public Health & Health Services Research, Central Denmark Region, Aarhus, Denmark; 8 Department of Obstetrics and Gynecology, Aarhus University Hospital, Aarhus, Denmark; 9 Maigaard Fertility Clinic, Aarhus, Denmark; 10 Centre for Integrated Register-Based Research Aarhus University, Aarhus, Denmark; 11 Danish Big Data Centre for Environment and Health (BERTHA), Aarhus University, Aarhus, Denmark

**Keywords:** Pregnancy loss, Drinking water nitrate, Nitrosatable drug exposure, Cohort study

## Abstract

**Background:**

Nitrate contamination is seen in drinking water worldwide. Nitrate may pass the placental barrier. Despite suggestive evidence of fetal harm, the potential association between nitrate exposure from drinking water and pregnancy loss remains to be studied. We aimed to investigate if nitrate in drinking water was associated with the risk of pregnancy loss.

**Methods:**

We conducted a nationwide cohort study of 100,410 pregnancies (enrolled around gestational week 11) in the Danish National Birth Cohort (DNBC) during 1996–2002. Spontaneous pregnancy losses before gestational week 22 were ascertained from the Danish National Patient Registry and DNBC pregnancy interviews. Using the national drinking water quality-monitoring database Jupiter, we estimated the individual and time-specific nitrate exposure by linking geocoded maternal residential addresses with water supply areas. The nitrate exposure was analyzed in spline models using a log-transformed continuous level or classified into five categories. We used Cox proportional hazards models to estimate associations between nitrate and pregnancy loss and used gestational age (days) as the time scale, adjusting for demographic, health, and lifestyle variables.

**Results:**

No consistent associations were found when investigating the exposure as a categorical variable and null findings were also found in trimester specific analyses. In the spline model using the continuous exposure variable, a modestly increased hazard of pregnancy loss was observed for the first trimester at nitrate exposures between 1 and 10 mg/L, with the highest.

adjusted hazard ratio at 5 mg/L of nitrate of 1.16 (95% CI: 1.01, 1.34). This trend was attenuated in the higher exposure ranges.

**Conclusion:**

No association was seen between drinking water nitrate and the risk of pregnancy loss when investigating the exposure as a categorical variable. When we modelled the exposure as a continuous variable, a dose-dependent association was found between drinking water nitrate exposure in the first trimester and the risk of pregnancy loss. Very early pregnancy losses were not considered in this study, and whether survival bias influenced the results should be further explored.

**Supplementary Information:**

The online version contains supplementary material available at 10.1186/s12940-022-00897-1.

## Introduction

A growing public concern has arisen over environmental substances, and how they may affect the human reproductive capacity [1–3]. As much as 30% of pregnancies result in pregnancy losses [4], but it remains unclear whether exposure to environmental pollutants (e.g. through drinking water) has harmful reproductive effects [5].

Nitrate can contaminate surface and groundwater due to intensive farming, which constitutes a man-made source of drinking water pollution [6–8]. Approximately 2–3% of the US and Western European populations are exposed to levels exceeding the drinking water standard at 50 mg/L nitrate (NO_3_^−^), which has been defined by the World Health Organization (WHO) [9]. The limit was set to protect newborns against methemeglobinemia, an acute life-threatening condition. In the 1960s, Schmitz suggested that high maternal methemeglobin levels could also cause pregnancy loss [10]. However, the drinking water standard does not address the potentially harmful effects of low-dose exposure to nitrate [11, 12].

Our knowledge on the potential adverse effects of low-dose exposure is based primarily on animal models, where nitrate has been suspected of endocrine disruptive, teratogenic and carcinogenic potential [13, 14]. The possible embryo-toxic effects from nitrate in humans are sparsely investigated, and previous studies have reported conflicting results and have methodological limitations [5, 15]. Nitrate is ingested from water and diet, has a short half-life and does not accumulate in the body [16]. It is mainly inactive and becomes biologically active after reduction to nitrite. Nitrite can subsequently react with amines and amides from diet to form *N*-nitroso compounds (NOCs). Nitrate and NOCs are water-soluble, thus exposing the fetus in utero [17]. The endogenous formation of NOCs may also be increased by use of nitrosatable drugs containing amides and amines [18], which are frequently used in pregnancy [19]. It is suspected that exposure to nitrosatable drugs in the presence of higher levels of nitrate may result in higher adverse health risk [20–22].

Even though pregnant women worldwide are exposed to nitrate every day, and pregnancy loss has devastating impact on the individual and society, only two studies have examined the association between drinking water nitrate and the risk of pregnancy loss in humans. A case control study of 1,677 women with exposure estimates at community level found no association at exposures below the drinking water standard [23], while a cluster analysis of four women proposed a harmful effect at levels exceeding the regulatory limits [24]. To our knowledge, the potential modifiable effect of nitrosatable drugs on nitrate in relation to pregnancy loss has not previously been studied.

We investigated the association between exposure to drinking water nitrate and pregnancy loss in the Danish National Birth Cohort (DNBC). The drinking water nitrate level in Denmark is generally below the regulatory limits, but even the exposure within this range has recently been suggested to adversely impact fetal growth [25]. We estimated trimester-specific hazard ratios while controlling for potential confounding by lifestyle factors. In supplemental analyses, we assessed whether nitrosatable drugs modified the association between drinking water nitrate and pregnancy loss.

## Methods

### Setting

Around two thirds of Denmark is agricultural land, with a high fertilization rate and livestock density [26]. Due to variations in geology and land use, groundwater and drinking water nitrate concentrations vary throughout the country [27, 28]. The Danish drinking water supply is decentralized and completely based on groundwater. The Danish population has high genetic homogeneity [29], and Danish residents are automatically enrolled in a free and universal health care system. All residents, including newborns and immigrants, are registered with a unique civil personal registration number (CPR number), which enables linkage of information across national registers [30].

### Study design and population

This nationwide cohort study include all 100,410 pregnancies enrolled in the DNBC in 1996–2002, and this data was linked with national registry data [31]. Pregnant women were invited to the DNBC by their general practitioner or midwife during antenatal visits in the first trimester, and the mean gestational age at enrolment was 11 (SD 3.4) completed weeks. To be included, the woman needed to be pregnant at the time of giving her written consent, to have residence in Denmark, to intend to complete the pregnancy and to be able understand and speak Danish. Around 50% of the general practitioners participated, and an estimated 60% of women accepted the invitation to enroll in the DNBC.

**Fig.1:**
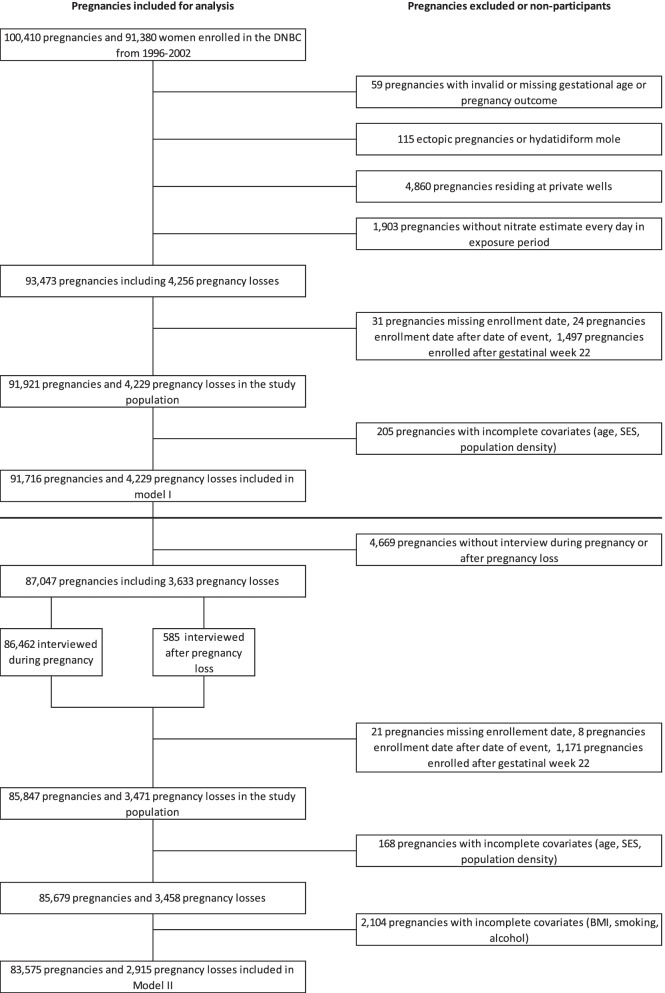
Flowchart of enrolled pregnancies in the DNBC 1996–2002 eligible for Cox analysis Model I and II

All enrolled women were invited to participate in four telephone interviews. The first interview was conducted around gestational week 16 and focused on reproductive history and lifestyle. If a pregnancy loss had occurred after enrolment and before the first regular interview, the woman was offered a “pregnancy loss” interview. This included the same baseline questions and additional questions regarding the cause of fetal death [32]. A total of 585 (0.7%) gave interview after a pregnancy loss. Women were eligible to participate in the DNBC multiple times if having consecutive pregnancies during the study period. We excluded women with unlikely enrolment dates or outcome dates (e.g. unrealistic due date, not physiologically plausible), women entering after end of follow-up and women with irrelevant outcome (i.e. extrauterine pregnancy), inaccurate exposure estimates or lack of any of the covariates (Fig. 1).

### Exposure estimates

Drinking water data originated from the national drinking water quality monitoring database.

Jupiter [33], where water samples analyzed by certified laboratories are registered. The exposure estimates for this study built on the approach developed and described by Schullehner et al. [6, 27, 34] and have been used in several observational studies of drinking water nitrate [11, 25, 35]. Using waterworks-specific annual nitrate levels is reasonable in Denmark, as drinking water is sourced from groundwater with no noteworthy seasonal variation and slow trends in concentration changes [27]. Individual level household exposure estimates with a resolution of one day were obtained through the geocoded residential history for every person registered in the Danish Civil Registration System [30], and these estimates were linked with water supply areas (WSA)[36]. From 1978, 98% of Danish residences were geocoded with geographical coordinates on municipality, road and house number with a resolution of one day [37]. The residential data was used to identify the WSA or private well supply of each household in the dataset by use of GIS analyses [6]. The annual nitrate concentration of the WSA was assigned to the household and weighted by drinking water production volume if a WSA had more than one supply source [6]. Thereby, it was possible to account for the specific residential pattern for each study participant, also if the woman moved during pregnancy. Nitrate concentrations measured at the waterworks correlate with the exposure at consumers’ taps [27, 28]. The highest detection limit in this data set was 1 mg/L. Analyses with detection limits above 1 mg/L were excluded. For computing exposure averages, measurements below the detection limit were imputed with 0.5 times the detection limit for the respective sample. Private wells are less consistently monitored, and information on nitrate levels is missing for approximately 50% [6]. Therefore, we included only individuals residing during the entire exposure period at an address registered at a public water supplier with at least one nitrate measurement taken within three years of the exposure window (approximately 94%). We imputed missing exposure if nitrate samples were available within three years. Imputation of missing years was necessary for around 10% of households each year, with almost all of these having an available sample in the previous or the following year; this was done by interpolation or using the closest observation for the earliest and latest year. The three-year period was accepted in consideration of the relatively stable nitrate concentrations over years. For the specific exposure windows in pregnancy, the exposure estimates were based on time-weighted average exposure. The nitrate exposure estimates took into account if the participant moved into a different residential area during the exposure window (9%) and considered yearly updated nitrate measurements if the pregnancy spanned over a calendar year.

Each woman’s nitrate exposure during pregnancy was calculated from the date of last menstrual period (LMP) to the date of pregnancy outcome or end of follow-up, whichever came first. For the analyses, end of follow-up was defined as gestational week 21 plus six days. For the sub-analyses assessing trimester-specific exposures, we calculated the exposure estimates up until gestational week 11 plus six days for the first trimester and from gestational week 12 up until gestational week 21 plus six days for the second trimester.

### Outcome

We defined the outcome of interest as a spontaneous intrauterine pregnancy loss before 22 completed weeks of gestation [38]. The enrolment criterion to the DNBC was ongoing pregnancy. DNBC participants were confirmed pregnant after clinical examination by their general practitioner. A subsequent loss after enrolment was documented in the DNBC with limited loss to follow-up (0.05% of pregnancies were excluded after enrolment due to registration errors of unlikely outcomes or gestational ages). Not all pregnancy losses are treated by healthcare professionals in Denmark. Therefore they might not be documented in the medical registries. For pregnancy losses not treated in hospitals, the pregnancy outcome was available from reports after enrolment by the pregnant woman in the DNBC interview [39]. Information on date of birth for all live-born children was obtained from the Danish Civil Registration System. Other pregnancy outcomes (ectopic pregnancy, hydatidiform mole, induced abortion, pregnancy loss and stillbirth) were available by linkage with the Danish National Patient Registry (DNPR) [39]. The DNPR contains national discharge data from 1977 onwards and categorizes diagnoses according to the International Classification of Diseases, 10^th^ revision. For each hospital contact, the diagnosis, treatment and potential surgical procedures are registered [40]. We estimated the gestational age of outcome of pregnancy from the first day in the LMP as reported by the women in the signed consent form to the DNBC.

### Covariates

Potential confounders and precision variables were identified a priori by review of the literature and by use of directed acyclic graphs (Figure S1) [41]. Maternal age at the time of pregnancy, socioeconomic factors, population density and lifestyle characteristics were adjusted for as listed in Table 1*.* Maternal age and population density were modelled as restricted cubic spline with four knots. Population density was included as a proxy for other neighbourhood and environmental factors and was defined as the number of people living 250 m or less from the residence of the woman at the time of pregnancy. Information on population density was based on the distance between geocoded address points from the Residence Database [42]. Socioeconomic status for each included pregnancy was available from Statistics Denmark and was based on the highest attained education (four groups) and occupation status at the start of pregnancy (eight groups) in accordance with the International Standard Classification of Education (ISCED) and International Standard Classification of Occupations (ISCO) codes [43, 44]. Besides drinking water, nitrate is found in foods (e.g. green vegetables, and processed meat) that also contain inhibitory antioxidants and vitamins reducing the potentially harmful contribution from the diet [45]. Additionally, we adjusted for lifestyle factors associated with dietary patterns. These were self-reported information collected from the DNBC interviews: pre-pregnancy body mass index (four groups), smoking (five groups) and alcohol (four groups). We also added information on all included pregnancies in the DNBC to restrict to primigravidae in a sensitivity analysis, with data on reproductive history developed by Chen et al. [46].

**Table 1:** Characteristics of the study population by nitrate concentration in drinking water (in five categories)

	**Nitrate concentration (mg/L)**				
**Characteristics**	** ≤ 1**	** > 1- ≤ 2**	** > 2- ≤ 5**	** > 5- ≤ 25**	** > 25**
Total pregnancies^a^, n (%)	27,413 (29.8)	25,753 (28.0)	24,285 (26.4)	10,449 (11.4)	4,021 (4.4)
Age at conception, mean (± SD)	29.56 (4.29)	29.90 (4.25)	29.86 (4.42)	29.44 (4.36)	29.32 (4.35)
Age at conception, n (%)					
< 20	282 (1.0)	224 (0.9)	261 (1.1)	129 (1.2)	48 (1.2)
≥ 20- < 25	3,461 (12.6)	2,744 (10.7)	2,860 (11.8)	1,443 (13.8)	568 (14.1)
≥ 25- < 30	11,653 (42.5)	10,616 (41.2)	9,719 (40.0)	4,340 (41.5)	1,705 (42.4)
≥ 30- < 35	9,049 (33.0)	9,030 (35.1)	8,364 (34.4)	3,386 (32.4)	1,280 (31.8)
≥ 35- ≤ 40	2,704 (9.9)	2,848 (11.1)	2,763 (11.4)	1,056 (10.1)	381 (9.5)
> 40	264 (1.0)	291 (1.1)	318 (1.3)	95 (0.9)	39 (1.0)
Highest education, n (%)					
Primary school	2,399 (8.8)	1,592 (6.2)	1,838 (7.6)	926 (8.9)	346 (8.6)
High school or vocational	11,449 (41.8)	8,721 (33.9)	8,833 (36.4)	4,495 (43.0)	1,600 (39.8)
Basic education	2,228 (8.1)	1,733 (6.7)	1,705 (7.0)	767 (7.3)	270 (6.7)
Middle education	8,838 (32.2)	8,548 (33.2)	7,732 (31.8)	3,290 (31.5)	1,361 (33.8)
Higher education or Ph.D	2,451 (8.9)	5,102 (19.8)	4,110 (16.9)	950 (9.1)	433 (10.8)
Missing	48 (0.2)	57 (0.2)	67 (0.3)	21 (0.2)	11 (0.3)
Occupation, n (%)					
Unemployed^b^	2,816 (10.3)	2,330 (9.0)	2,311 (9.5)	1,211 (11.6)	471 (11.7)
Student	2,036 (7.4)	3,130 (12.2)	2,305 (9.5)	813 (7.8)	378 (9.4)
Employee unspecified income	2,066 (7.5)	1,671 (6.5)	1,637 (6.7)	779 (7.5)	287 (7.1)
Employee with low income	10,090 (36.8)	7,344 (28.5)	7,747 (31.9)	3,895 (37.3)	1,390 (34.6)
Employee with middle income	6,371 (23.2)	6,149 (23.9)	5,613 (23.1)	2,255 (21.6)	887 (22.1)
Chief executive or high income	3,026 (11.0)	4,221 (16.4)	3,770 (15.5)	1,147 (11.0)	469 (11.7)
Owner of business	501 (1.8)	496 (1.9)	474 (2.0)	192 (1.8)	58 (1.4)
Other	507 (1.8)	412 (1.6)	> 422(1.7)	157 (1.5)	81 (2.0)
Missing	0 (0.0)	0 (0.0)	< 5 (.)	0 (0.0)	0 (0.0)
Population density^c^, mean (± SD)	424.88 (488.63)	1215.16 (1382.67)	1137.34 (1306.25)	624.18 (874.46)	592.85 (717.16)
Smoking in pregnancy (cigarettes/day), n (%)					
Non-smoker	18,937 (69.1)	17,905 (69.5)	16,364 (67.4)	7,038 (67.4)	2,781 (69.2)
Smoked in early pregnancy	2,061 (7.5)	2,460 (9.6)	2,280 (9.4)	879 (8.4)	295 (7.3)
1–9	2,365 (8.6)	2,027 (7.9)	2,045 (8.4)	965 (9.2)	320 (8.0)
10–14	1,172 (4.3)	980 (3.8)	1,066 (4.4)	538 (5.1)	210 (5.2)
> 15	737 (2.7)	576 (2.2)	712 (2.9)	349 (3.3)	129 (3.2)
Missing	2,141 (7.8)	1,805 (7.0)	1,818 (7.5)	680 (6.5)	286 (7.1)
Weekly alcohol consumption (units/week), n (%)					
0	13,860 (50.6)	12,911 (50.1)	12,677 (52.2)	5,613 (53.7)	2,121 (52.7)
0.5–3.5	10,866 (39.6)	10,468 (40.6)	9,276 (38.2)	3,940 (37.7)	1,539 (38.3)
4–7	489 (1.8)	517 (2.0)	447 (1.8)	181 (1.7)	> 65 (1.6)
≥ 7.5	39 (0.1)	36 (0.1)	59 (0.2)	20 (0.2)	< 5 (.)
Missing	2,159 (7.9)	1,821 (7.1)	1,826 (7.5)	695 (6.7)	288 (7.2)
Body mass index (kg/m2), n (%)					
< 18.5	1035 (3.8)	1,097 (4.3)	1,098 (4.5)	439 (4.2)	166 (4.1)
18.5–25	16,262 (59.3)	16,761 (65.1)	15,371 (63.3)	6,206 (59.4)	2,350 (58.4)
25–30	5,238 (19.1)	4,096 (15.9)	4,011 (16.5)	2,051 (19.6)	797 (19.8)
> 30	2,360 (8.6)	1,607 (6.2)	1,613 (6.6)	930 (8.9)	369 (9.2)
Missing	2,518 (9.2)	2,192 (8.5)	2,192 (9.0)	823 (7.9)	339 (8.4)
Gravidity, n (%)					
Primigravidae	9,604 (35.0)	9,988 (38.8)	8,971 (36.9)	3,632 (34.8)	1,517 (37.7)
Multigravidae	> 17,803(64.9)	15,765 (61.2)	> 15,308 (63.0)	> 6,811 (65.2)	2,504 (62.3)
Missing	< 5 (.)	0 (0.0)	< 5 (.)	< 5 (.)	0 (0.0)
Parity, n (%)					
Nulliparous	12,607 (46.0)	13,825 (53.7)	12,630 (52.0)	4,901 (46.9)	1,998 (49.7)
Multiparous	> 14,800 (54.0)	11,928 (46.3)	> 11,649 (48.0)	> 5,542 (53.1)	2,023 (50.3)
Missing	< 5 (.)	0 (0.0)	< 5 (.)	< 5 (.)	0 (0.0)
Previous spontaneous pregnancy loss, n (%)					
0	21,941 (80.0)	20,707 (80.4)	19,547 (80.5)	8,394 (80.3)	3,212 (79.9)
1–2	5,283 (19.3)	4,917 (19.1)	4,564 (18.8)	1,977 (18.9)	776 (19.3)
> 3	> 183 (0.7)	129 (0.5)	> 168 (0.7)	> 72 (0.7)	33 (0.8)
Missing	< 5 (.)	0 (0.0)	< 5 (.)	< 5 (.)	0 (0.0)
Nitrosatable drug in pregnancy, n (%)					
Yes	4,321 (15.8)	3,726 (14.5)	3,795 (15.6)	1,682 (16.1)	650 (16.2)
No	23,082 (84.2)	22,027 (85.5)	> 20,484 (84.4)	> 8,761 (83.9)	3,371 (83.8)
Missing	10 (0.0)	0 (0.0)	< 5 (.)	< 5 (.)	0 (0.0)

According to local regulations, single values smaller than five corresponding to participants in the study may not be reported (GDPR, Regulation (EU), 2016/679 of 25 May 2018). In case of numbers below five, pseudo-numbers were estimated as the value nearest to the actual count > or < five

^a^ *n* = 91,921

^b^ Unemployed: social security benefits, disability pension or state education grant

^c^ Population density below 250 m

### Effect modifier

To stratify by drug nitrosatability in a supplemental analysis, we identified the specific Anatomical Therapeutic Chemical (ATC) codes with further adjustment to the Danish setting; this approach has been described in detail elsewhere [19, 47]. Prescribed drugs during pregnancy were identified by linkage to the National Prescription Registry (NPR) through the use of ATC codes [48]. The NPR holds precise data on redeemed prescriptions, with an estimated reliability of > 97% [49]. However, over-the-counter drugs are not included in the NPR. Women prescribed nitrosatable drugs from the date of LMP to the outcome date or end of follow-up were categorized as exposed from the date of redemption. Only the first prescribed drug was counted. To capture women with drug redemption in the periconceptual period, we also included a woman as exposed if she had redeemed a nitrosatable drug from 14 days prior to the date of LMP. Women without any redemptions of nitrosatable drugs from 14 days prior the date of LMP until the outcome date or end of follow-up were categorized as unexposed.

### Statistical analyses

We used Cox proportional hazard models to estimate hazard ratios (HRs) with 95% confidence intervals (CIs) for the association between drinking water nitrate and pregnancy loss.

Drinking water nitrate concentrations were categorized into five a priori defined groups as previously described: ≤ 1 mg/L, > 1- ≤ 2 mg/L, > 2- ≤ 5 mg/L, > 5- ≤ 25 mg/L and > 25 mg/L [25]. The lowest reference category comprised exposures below the detection limit in the dataset (≤ 1 mg/L). Further, for the analyses of continuous exposure, the nitrate concentration distribution was not linear and a log-transformed continuous variable was modelled in restricted cubic splines with four knots assigned to the 5th, 35th, 65th and 95th percentile [50].

The Cox model enabled delayed entry at recruitment to the DNBC, and the underlying time variable was gestational age in days. Induced abortions were considered competing risks. Follow-up was terminated at gestational age 21 weeks plus six days or pregnancy loss, whichever came first. Overall hazards up to 22 weeks of gestation were supplemented with analysis on trimester specific hazards from date of LMP to gestational age 11 weeks plus six days (referred to as “first trimester”) and further from gestational week 12 to gestational age 21 weeks plus six days (referred to as “second trimester”).

Dependency of pregnancies by the same woman was accounted for by robust standard error.

The proportional hazard assumption for the categorical variables was evaluated by log–log plots and continuous variables by restricted cubic splines. The log–log plots showed a good approximation, and the proportional hazards assumption was accepted.

Specific adjusted hazard ratios and CIs of pregnancy loss were read in Stata for the splines with water nitrate exposure (log scale) in pregnancy, with 1 mg/L as the reference.

For pregnancy losses with missing gestational age of outcome (*n* = 64), we estimated the event date by simple imputation. We imputed a median gestational age of early pregnancy loss (nine weeks), as reported in the reproductive history of DNBC women [46]. In the main model, 40 pregnancies with imputed date of event entered the analysis.

### Sensitivity analyses

We performed a number of sensitivity analyses to test the robustness of our findings and to explore potential exposure misclassification due to unknown nitrate levels or selection bias.

The first sensitivity analysis was a basic model adjusting for maternal age at the time of pregnancy, socioeconomic factors and population density, leaving out lifestyle factors. The second sensitivity analysis excluded all pregnancies during which the woman had moved to a different geographical location in the exposure window (*n* = 8,444) to account for potential misclassification of exposure. Private wells have higher nitrate concentrations, yet limited monitoring, compared to public water supplies. Therefore, the third sensitivity analysis included women residing at locations with private wells with nitrate information at any time during the exposure period. This resulted in 94,317 pregnancies eligible for analysis. In a fourth sensitivity analysis, we performed a logistic regression analysis with dichotomized outcome categorization. Previous reproductive history was not adjusted for in the main analysis because adjustment for past pregnancy history may induce collider stratification bias [51]. To address this potential bias, we conducted a fifth sensitivity analysis, which restricted the study population to primigravidae (*n* = 33,616) [52]. We defined primigravidae as women who were pregnant for the first time and multigravidae as women with more than one pregnancy.

From 2004, the cut-off between pregnancy loss and stillbirth was changed from 28 to 22 completed weeks of gestation in Denmark. Thus, in a sixth sensitivity analysis, we investigated if follow-up until gestational week 28 changed the results by using exposure calculations adapted to this time interval (from date of LMP up until GA 27 weeks plus six days).

To investigate potential effect modifications by age, we conducted a seventh sensitivity analysis stratified by age (≤ 25 year and > 25 years).

To examine whether exposure to nitrosatable drugs modified the association, we modelled a Cox regression analysis in a supplemental analysis, where the dataset was split into specific exposure records according to date of drug redemption. Thereby, a woman was categorized as unexposed up until the date of redemption and as exposed thereafter. Drinking water nitrate was categorized into five categories as previously described. Test for effect modification was performed by Wald test.

Modelling of the exposure was performed by use of R (version 3.6; R Development Core Team), whereas the analyses were performed in Stata, version 15.0 (StataCorpLP, College Station, TX, USA).

## Results

The study population consisted of 91,921 pregnancies and 4,229 (4.6%) pregnancy losses (Fig. 1). The exposure distribution was right skewed (median 1.81 mg/L (95% prediction interval: (0.17–18.74 mg/L)), and 4.4% of pregnancies were exposed to nitrate levels above 25 mg/L. Table 1 displays the characteristics of the study population. The proportion of missing data was evenly distributed across the exposure categories (Table 1).

When studying the categorical exposure, we found no evidence of an association between drinking water nitrate and pregnancy loss (Table 2). When exploring the timing of exposures, we found that the hazard ratios were slightly higher in the first trimester and lower in the second trimester with overlapping 95% CIs. Further, in the categorical analyses, no dose–response relationship was found.

**Table 2:** Adjusted hazard ratios (HR) and 95% confidence intervals (CI’s) of pregnancy loss associated with drinking water nitrate exposure in pregnancy

	Pregnancy to week 22^a^			First trimester^b^			Second trimester^c^		
	*n* = 83,575 (2,915 pregnancy losses)			*n* = 53,082 (1,481 pregnancy losses)			*n* = 82,004 (1,352 pregnancy losses)		
NO_3_^-^ (mg/L)	Pregnancies (n (%))	Pregnancy losses (n)	aHRd (95% CI)	Pregnancies (n (%))	Pregnancy losses (n)	aHR^d^ (95% CI)	Pregnancies (n (%))	Pregnancy losses (n)	aHR^d^ (95% CI)
≤ 1	24,829 (29.7)	831	Ref (1)	15,428 (29.1%)	397	Ref (1)	24,717 (30.1)	423	Ref (1)
> 1- ≤ 2	23,474 (28.1)	829	0.97 (0.88, 1.07)	15,263 (28.8)	433	0.98 (0.86, 1.14)	22,894 (27.9)	363	0.91 (0.77, 1.06)
> 2- ≤ 5	22,013 (26.3)	781	1.00 (0.90, 1.10)	13,983 (26.3)	408	1.03 (0.89, 1.19)	21,492 (26.2)	351	0.95 (0.82, 1.10)
> 5- ≤ 25	9,586 (11.5)	346	1.04 (0.92, 1.18)	6,011 (11.3)	176	1.10 (0.92, 1.31)	9,229 (11.3)	156	0.98 (0.81, 1.17)
> 25	3,673 (4.4)	128	0.97 (0.81, 1.17)	2,397 (4.5)	67	1.03 (0.80, 1.34)	3,672 (4.5)	59	0.92 (0.70, 1.21)

Model was fitted using robust standard error to control for non-independence of pregnancies by the same woman

*NO*_*3*_^*-*^ Nitrate concentration in drinking water, *CI* Confidence interval, *Ref* Reference

^a^ LMP to GA 21 weeks plus six days

^b^ LMP to GA 11 weeks plus six days

^c^ GA 12 weeks to GA 21 weeks plus six days

^d^ Adjusted for maternal age, education, occupation, population density, BMI, smoking and alcohol

**Fig. 2:**
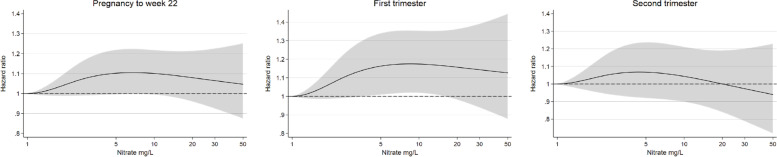
Adjusted hazard ratios of pregnancy loss by drinking water nitrate exposure (log scale) in pregnancy with 1 mg/L as reference. Exposures below the highest detection limit 1 mg/L and above 50 mg/L are not shown, but included in the model. Grey scale areas represent the CI. Splines were adjusted for age, education, occupation, population density, BMI, smoking and alcohol. Robust standard errors accounted for dependencies between pregnancies by the same woman

In Fig. 2, splines describe the associations between exposure concentrations and pregnancy loss. The splines showed a weak dose-related increase in the hazards of pregnancy loss up to concentrations of 5 mg/L, after which the curve plateaued and finally decreased for the highest exposure concentrations above 10 mg/L, however with wide CIs. The highest hazard of pregnancy loss was in the first trimester analysis with adjusted hazard ratio of 1.16 (95% CI: 1.01, 1.34) at 5 mg/L nitrate.

The results of the sensitivity analyses indicated robustness of the main results (Table S1 and Figure S2). Restricting to women who resided at the same water supply throughout pregnancy replicated the main results (Table S2). Reintroducing private well users in the model also revealed adjusted hazard ratios around 1 (Table S3). Results were also comparable in the logistic regression model (Table S4), in the sensitivity analysis restricted to primigravidae (Table S5) and when extending the follow-up period to gestational week 28 (Table S6). In younger women below 25 years, the hazard ratios increased slightly with nitrate exposure, however with CIs including the null. Women above 25 years of age showed similar results as the main findings (Table S7).

Of the included pregnancies, 16% redeemed nitrosatable drugs in early pregnancy (Table 1). We found no evidence of effect modification on the multiplicative scale by use of nitrosatable drugs in any of the drinking water nitrate groups and increased hazard of pregnancy loss (Table S8). The association was homogeneous throughout nitrate groups between women who had prescribed a nitrosatable drug in pregnancy and women without redemptions.

## Discussion

In this nationwide cohort study, no association was found between drinking water nitrate and the risk of pregnancy loss in the categorical analyses. When we modelled the exposure as a continuous variable, the risk of pregnancy loss was as high as up to 10–16% in the group of pregnancies exposed to 1–10 mg/L nitrate in the first trimester analyses, but it was lower in the highest exposures above 10 mg/L with 1 mg/L as reference, and the same trend in results was found in the sensitivity analyses omitting adjustment of lifestyle factors. Regardless of the Cox model or exposure period used, we consistently observed higher risk with low dose exposures throughout pregnancy when we modelled the exposure as a continuous variable, however not statistically significant. This was most pronounced in the first trimester, which may suggest that the timing and the dose of nitrate exposure influence the risk of pregnancy loss. Stratifying on age indicated that women below 25 years might have higher hazard ratio compared to women above 25 years. This could be due to higher susceptibility for unknown biological reasons or chance finding. Our results did not indicate effect modification by nitrosatable drugs.

### Comparison with other studies

Animal studies have suggested longer days to litter, reduced number of offspring, cycle irregularities and higher rates of fetal death among female mice and cattle exposed to nitrate [53–57]. Impaired semen concentration, motility and morphology have been reported among exposed male rats and mice [58, 59]. In observational studies among humans, drinking water nitrate has been associated with neonatal death [60], intrauterine growth retardation [25, 61], prematurity [62], very low birth weight [63, 64] and congenital malformations [65, 66] at exposure levels below the drinking water standard. These outcomes potentially share underlying mechanisms with pregnancy loss. However, previous results did not suggest increased risk of pregnancy loss at nitrate exposures of 0.1–5.5 mg-N/L (corresponding to 0.44–24.3 mg/L) [23]. In this case–control study, drinking water samples were collected from community level drinking water sources, and pregnancy loss was reported for women admitted to hospital. Yet, given the limitations of the study design and potential exposure and outcome misclassifications, the validity of previous studies has been questioned [15].

We hypothesized that nitrosatable drugs might serve as effect modifiers for the association between drinking water nitrate and pregnancy loss. Prenatal nitrosatable drug exposure has previously been associated with congenital malformations, preterm birth and stillbirth [22, 47, 67, 68]. Further, nitrosatable drugs and nitrate intake in combination has been associated with neural tube defects and preterm birth [69, 70].

### Methodological considerations

To our knowledge, this is the first study in this field to include a large population with highly accurate measurements of drinking water nitrate at household level, valid data on pregnancy loss and comprehensive covariate information. The time-specific information on conception, entry and outcome, as well as the date-specific exposure estimates and drug redemption dates are unique. This detailed data enabled analyses of time-dependent associations with almost complete follow-up, and censoring because of loss to follow-up was no concern.

Several potential limitations should be considered. The DNBC is estimated to cover 31% of all pregnancies in Denmark during the study period [71]. A lower participation rate was found among women with lower income or education and younger age at the time of pregnancy. Therefore, non-participation needs to be addressed. As it is not well-established in epidemiological literature to apply quantitative bias analyses to time-to-event data, a qualitative assessment was made [72]. It is reassuring that the selective participation in the DNBC (associated with higher age and higher socioeconomic position) was not found to induce considerable bias by comparing ORs in DNBC to ORs in a sample of the source population for the participating women in the DNBC [73]. Associations of i) in vitro fertilization and preterm birth, ii) smoking and small for gestation age, iii) BMI and stillbirth were compared in the two study populations, and the findings of minor risk of bias were reassuring, specifically in consideration of our exposure and outcome.

In the DNBC, 4.6% of the included pregnancies resulted in a pregnancy loss. Pregnancy loss is a common outcome of pregnancy [4], and only around 50% of pregnancy losses are clinically recognized. A Danish study including national medical register data found 5–10% registered spontaneous pregnancy losses in women of fertile age [74]. In our study, fetal losses occurring before enrolment in the DNBC (on average at gestational week 11) would not be captured. The medical registries and the DNBC documented pregnancy losses that led to a consultation with specialist healthcare services or appeared in the pregnancy interviews after enrolment. If nitrate exposure affects fetal loss starting from early gestation, the exposure would directly impact the probability of being selected into the cohort. Conditioning on selected fetuses surviving long enough for their mothers to be included in the study may induce a collider bias. Our previous simulation study using the DNBC has shown that a bias towards the negative direction could occur when a toxic exposure affects the risk of fetal loss, and the statistical analyses were conducted among the surviving fetuses [75]. This bias could also potentially have influenced our analyses if nitrate associated pregnancy loss occurred prior to the DNBC enrolment, especially for the higher exposure range, where a downward exposure and outcome trend were found. In addition, the effect estimates also seem to diminish in the analyses among those surviving through the second trimester. Therefore, it needs to be investigated whether survival bias affected our findings and biased the results towards the null, for example by exploring the influence of nitrate exposure on fertility and/or the incidence of early pregnancy losses in another study sample.

The water supply exposure estimates at residence level were used as a measure of individual bioavailability, as we were not able to provide data for nitrate in diet or individual samples of the bioavailability. Therefore, we cannot rule out exposure misclassification and expect differences in individual consumption and metabolism to be non-differential, which may have biased the results towards the null. However, during the study period in Denmark, people primarily consumed tap water, and the intake of bottled water was the lowest (20.5 L per year in 2012) in Denmark compared with other European countries [76, 77] and the US [78]. The exposure estimates were collected independent of the outcome, and they rely on day-specific residential information for each study participant. The water samples were analyzed at certified laboratories, and the estimates took water production volume into account. In a nationwide Danish study, drinking water nitrate concentrations at the waterworks were highly correlated with concentrations at the consumers’ taps (R^2^ = 0.98) and showed no seasonal variations, indicating high validity of the available exposure data used in our study [27]. Groundwater is the only source of Danish drinking water, resulting in negligible seasonal variation and stable concentrations from the same aquifer over several years with gradual changes [6, 27]. We calculated the average estimate of exposure from LMP to date of event, and we relied on the number of days in which the woman resided at a specific address in case she moved during pregnancy. The sensitivity analysis restricted to women with the same address in the entire study period did not change our results.

For the pregnancies excluded due to poor exposure quality (users at private wells or no information on exposure every day in the study period), we considered the risk of selection bias. For the exposure estimates of the excluded pregnancies, we could not calculate a valid exposure assessment as they were excluded due to missing nitrate estimates. However, we found the same risk of pregnancy loss among the excluded pregnancies as among the included pregnancies (4.6% for private wells and 4.3% for missing exposure), which limits the risk of selection bias of these exclusions.

The comprehensive data recorded in the Danish registries and the DNBC allowed us to account for a range of important potential confounders, such as socioeconomic status and lifestyle-related variables. Covariates selected from the Danish national registries had high validity and completeness. Information on lifestyle may be remembered differently for women after a pregnancy loss than for women still pregnant at the time of interview. However, only 585 (16%) women with pregnancy loss provided information about lifestyle after the pregnancy ended.

We have adjusted for lifestyle factors associated with dietary patterns [79] in our analyses, but residual confounding and unmeasured confounding because of specific dietary factors cannot be ruled out. Diet is complex in studies of nitrate in drinking water as the expected harmful mechanisms of nitrate are connected to the amount of NOCs formed, and NOC formation depends on the metabolites ingested from drinking water, diet, drugs and endogenous factors [5, 80]. The net endogenous formation of NOCs depends on both the presence of inhibitors (e.g. vitamin C and E) and nitrosation precursors (e.g. red meat, nitrosatable drugs), the acid environment in the stomach and the concentration of components involved [5, 15]. We did not have nitrate from diet available for this project. We considered SES and lifestyle factors as proxies for unmeasured factors associated with dietary nitrate. As intake of processed and cured meat contains exogenous NOCs, and a meat-rich diet may be associated with income and vegetables rich in nitrate, SES also influences the amount of NOCs and nitrate in diet [79]. SES and lifestyle factors such as BMI, smoking and alcohol are, however, not a perfect proxy for a particular dietary pattern.

When considering adverse effects of nitrate, confounding by other environmental factors also needs to be considered. Pollution of the groundwater with pesticides might coincide with nitrate containing groundwater due to leaching from agricultural activities [81, 82]. We do not have data on pesticides, but our results were adjusted for population density as a proxy for environmental factors associated with geographical differences and urbanicity in Denmark.

A limitation of the supplemental analysis on effect modification by nitrosatable drugs was the low statistical power. We used nitrosatable drug redemption as a measure for nitrosatable drug intake. This does not include over-the-counter medications, which may have biased our results towards the null. Further, when using drug redemption as a measure of drug intake, compliance during pregnancy needs to be questioned. It is also a limitation in our study that we were unable to account for comorbidity as confounding by indication, which could have biased results.

## Conclusion

In this Danish cohort study with individual level exposure and outcome assessments, the findings do not clearly support an association between nitrate and pregnancy loss. However, we found some evidence to suggest that exposure to drinking water nitrate below the drinking water standard in the first trimester is associated with increased risk of pregnancy loss. As nitrate in drinking water is widespread, the finding could have important public health implications. We cannot rule out biased results towards the null due to potential exposure misclassification and selection bias. Therefore, our results need to be confirmed in other populations, and such replication studies would benefit from including exposure data on individual bioavailability and outcomes of infertility and early pregnancy losses.

## Supplementary Information

**Additional file 1.** 

## Data Availability

The analyses for this study were performed at Statistics Denmark.

## Abbreviations

NO_3_^−^: Nitrate
NO_2_^−^: Nitrite
NOCs: *N*-Nitroso compounds
CIs: Confidence intervals,
HRs: Hazard ratios
aHRs: Adjusted hazard ratios
