# Supplementary material for: Drinking water nitrate and risk of pregnancy loss: a nationwide cohort study
Source: Environ Health. 2022 Sep 16;21:87. doi: 10.1186/s12940-022-00897-1 (PMC9479399; doi:10.1186/s12940-022-00897-1)
Supplement: Supplementary file 1 — Additional file 1. [file 12940_2022_897_MOESM1_ESM.docx]

**Figure S1**: Directed acyclic graphs (DAGs) used to select covariates for the basic model and main model.

Basic model


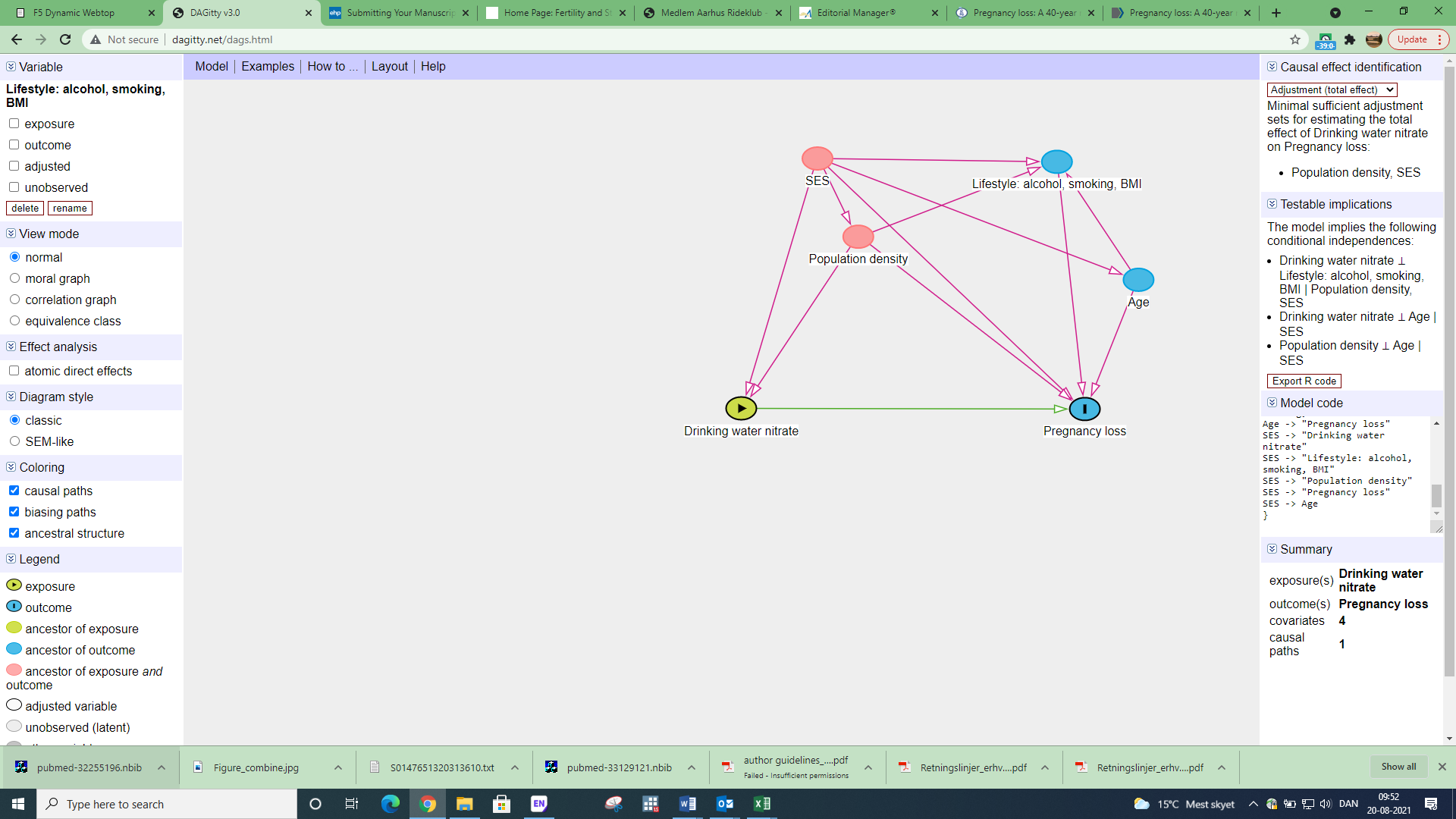


Main model


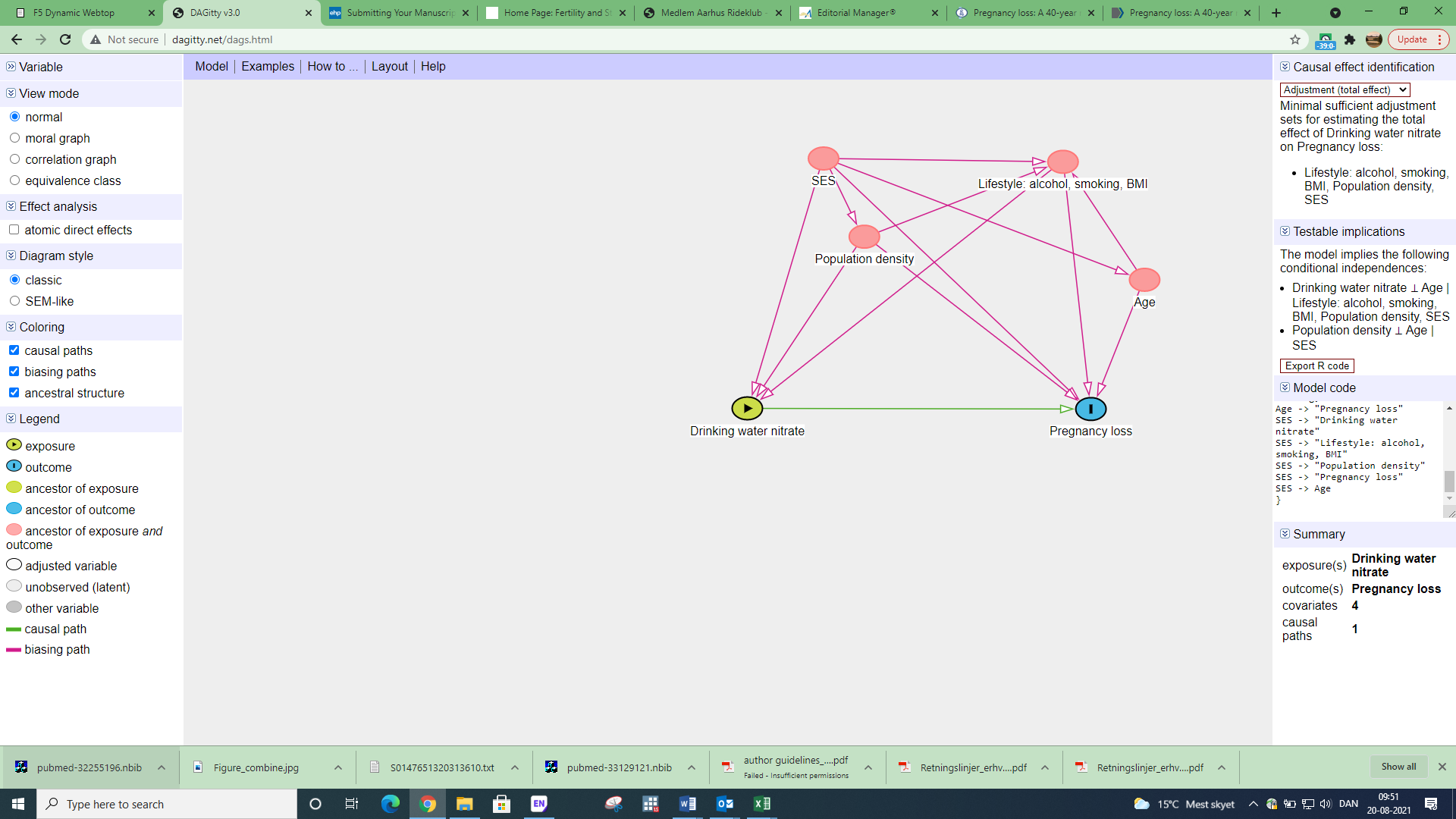


Textor J, van der Zander B, Gilthorpe MS, Liskiewicz M, Ellison GT. 2016. Robust causal inference using directed acyclic graphs: The r package 'dagitty'. Int J Epidemiol 45:1887-1894, PMID:28089956, <https://doi.org/10.1093/ije/dyw341>.

| **Table S1.** Basic adjustment: Adjusted hazard ratios (aHR) and 95% confidence intervals (CI's) of pregnancy loss associated with drinking water nitrate exposure in pregnancy. | | | | | | | | | | | | | | | |  |
| --- | --- | --- | --- | --- | --- | --- | --- | --- | --- | --- | --- | --- | --- | --- | --- | --- |
|  |  | Pregnancy to week 22^a^ | | |  | First trimester^b^ | | | |  | |  | Second trimester^c^ | | | |
|  |  | n=91,716 (4,212 pregnancy losses) | | |  | n=58,259 (2,254 pregnancy losses) | | |  | | n=89,249 (1,838 pregnancy losses) | | | | | |
| NO_3_⁻ (mg/L) |  | Pregnancies (n (%)) | Pregnancy losses (n) | aHRd (95% CI) |  | Pregnancies (n (%)) | Pregnancy losses (n) | aHR^d^  (95% CI) | |  | |  | Pregnancies (n (%)) | Pregnancy losses (n) | aHR^d^ (95% CI) | |
| ≤ 1 |  | 27,365 (29.8) | 1,215 | Ref (1) |  | 16,998 (29.2) | 617 | Ref (1) |  | | 27,022 (30.3) | | | 577 | Ref (1) | |
| >1- ≤ 2 |  | 25,696 (28.0) | 1,206 | 0.97 (0.87, 1.06) |  | 16,725 (28.7) | 659 | 1.00 (0.90, 1.13) |  | | 24,848 (27.8) | | | 505 | 0.93 (0.82, 1.05) | |
| > 2- ≤ 5 |  | 24,217 (26.4) | 1,144 | 1.00 (0.92, 1.09) |  | 15,383 (26.4) | 627 | 1.04 (0.93, 1.16) |  | | 23,431 (26.3) | | | 478 | 0.94 (0.83, 1.07) | |
| > 5- ≤ 25 |  | 10,428 (11.4) | 468 | 0.97 (0.88, 1.08) |  | 6,532 (11.2) | 250 | 1.03 (0.89, 1.19) |  | | 9,973 (11.2) | | | 204 | 0.95 (0.81, 1.11) | |
| > 25 |  | 4,010 (4.4) | 179 | 0.94 (0.89, 1.10) |  | 2,621 (4.5) | 101 | 1.01 (0.82, 1.25) |  | | 3,975 (4.5) | | | 74 | 0.86 (0.67, 1.09) | |
| Note: Model was fitted using robust standard error to control for nonindependence of pregnancies by the same woman. NO_3_⁻, nitrate concentration in drinking water; CI, confidence interval; Ref, reference. | | | | | | | | | | | | | | | | |
| ^a^LMP to GA 21 weeks plus 6 days. | | | | | | | | | | | | | | | | |
| ^b^LMP to GA 11 weeks plus 6 days. | | | | | | | | | | | | | | | | |
| ^c^GA 12 weeks to GA 21 weeks plus 6 days. | | | | | | | | | | | | | | | | |
| ^d^Adjusted for maternal age, education, occupation and population density. | | | | | | | | | | | | | | | | |

**
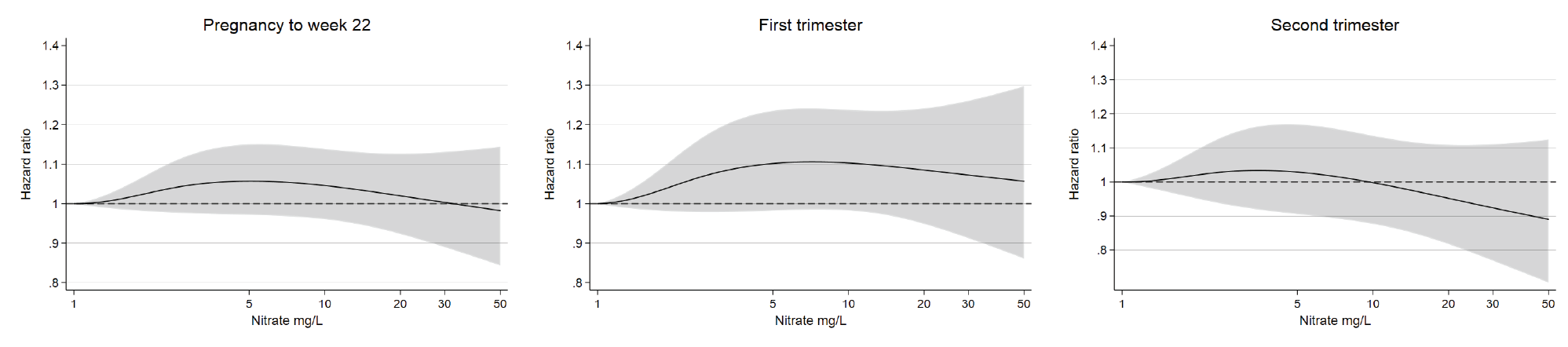
**

**Figure S2**: Adjusted hazard ratios of pregnancy loss by drinking water nitrate exposure (log scale) in pregnancy with 1 mg/L as reference. Exposures below the highest detection limit 1 mg/L and above 50 mg/L are not shown, but included in the model. Grey scale areas represent the CI. Splines (basic model) were adjusted for maternal age, maternal education, maternal occupation and population density. Robust standard errors accounted for dependencies between pregnancies by the same woman.

| **Table S2.** Cox regression for the association between pregnancy loss until gestational week 22 and drinking water nitrate including risk factors and confounders restricted to women residing at the same address in exposure period. | | | |
| --- | --- | --- | --- |
|  |  |  |  |
|  | n=83,272 (3,978 pregnancy loss) | | |
| NO_3_⁻ (mg/L) | n (%) | Pregnancy loss (n) | aHR (95% CI) |
| Categorical |  |  |  |
| ≤ 1 | 25,266 (30.3) | 1,154 | Ref (1) |
| >1- ≤ 2 | 23,266 (27.9) | 1,138 | 0.98 (0.89, 1.06) |
| > 2- ≤ 5 | 21,696 (26.1) | 1,078 | 1.01 (0.92, 1.10) |
| > 5- ≤ 25 | 9,371 (11.3) | 437 | 0.98 (0.88, 1.10) |
| > 25 | 3,673 (4.4) | 171 | 0.95 (0.81, 1.11) |
| Note: Adjusted for maternal age, maternal education, maternal occupation, population density. Model was fitted using robust standard error to control for non-independence of pregnancies by the same woman. NO_3_⁻, nitrate concentration in drinking water; CI, confidence interval; Ref, reference. | | | |

| \| **Table S3.** Cox regression for the association between pregnancy loss until gestational week 22 and drinking water nitrate including risk factors and confounders. Study population including private well users. \| \| \| \| \| --- \| --- \| --- \| --- \| \| \|  \| n=94,317 (4,337 pregnancy loss) \| \| \| \| NO_3_⁻ (mg/L) \| n (%) \| Pregnancy loss (n) \| aHR (95% CI) \| \| Categorical \|  \|  \|  \| \| ≤ 1 \| 28,161 (29.9) \| 1,263 \| Ref (1) \| \| >1- ≤ 2 \| 25,840 (27.4) \| 1,213 \| 0.97 (0.89, 1.05) \| \| > 2- ≤ 5 \| 24,397 (25.9) \| 1,152 \| 0.99 (0.91, 1.08) \| \| > 5- ≤ 25 \| 10,969 (11.6) \| 491 \| 0.97 (0.87, 1.07) \| \| > 25 \| 4,950 (5.3) \| 218 \| 0.95 (0.82, 1.10) \| \| Note: Adjusted for maternal age, maternal education, maternal occupation and population density. Model was fitted using robust standard error to control for nonindependence of pregnancies by the same woman. NO_3_⁻, nitrate concentration in drinking water; CI, confidence interval; Ref, reference \| \| \| \| |
| --- | --- | --- | --- | --- | --- | --- | --- | --- | --- | --- | --- | --- | --- | --- | --- | --- | --- | --- | --- | --- | --- | --- | --- | --- | --- | --- | --- | --- | --- | --- | --- | --- | --- | --- | --- | --- | --- | --- | --- | --- |
|  |

| **Table S4.** Logistic regression for the association between pregnancy loss until gestational week 22 and drinking water nitrate including risk factors and confounders. | | | |  |
| --- | --- | --- | --- | --- |
|  |  |  |  |  |
|  | n=91,716 (4,229 pregnancy loss) | | |  |
| NO_3_⁻ (mg/L) | n (%) | HR | aHR (95% CI) |  |
| Categorical |  |  |  |  |
| ≤ 1 | 27,365 (29.8) | Ref (1) | Ref (1) |  |
| >1- ≤ 2 | 25,696 (28.0) | 1.05 (0.97, 1.13) | 1.00 (0.92, 1.08) | |
| > 2- ≤ 5 | 24,217 (26.4) | 1.06 (0.98, 1.15) | 1.00 (0.92, 1.09) |  |
| > 5- ≤ 25 | 10,428 (11.4) | 1.02 (0.91, 1.13) | 1.01 (0.97, 1.12) |  |
| > 25 | 4,010 (4.4) | 1.03 (0.88, 1.12) | 1.02 (0.87, 1.19) |  |
| Note: Adjusted for maternal age, maternal education, maternal occupation, population density. Model was fitted using robust standard error to control for nonindependence of pregnancies by the same woman. NO_3_⁻, nitrate concentration in drinking water; CI, confidence interval; Ref, reference. | | | |  |

| **Table S5.** Cox regression for the association between pregnancy loss until gestational week 22 and drinking water nitrate including risk factors and confounders. Restricted to primigravida. | | | |
| --- | --- | --- | --- |
|  |  |  |  |
|  | n=33,616 (1,441 pregnancy loss) | | |
| NO_3_⁻ (mg/L) | n (%) | pregnancy loss, (n) | aHR (95% CI) |
| Categorical |  |  |  |
| ≤ 1 | 9,586 (28.5) | 388 | Ref (1) |
| >1- ≤ 2 | 9,957 (29.6) | 458 | 1.04 (0.99, 1.20) |
| > 2- ≤ 5 | 8,940 (26.6) | 399 | 1.04 (0.90, 1.21) |
| > 5- ≤ 25 | 3,623 (10.8) | 133 | 0.86 (0.71, 1.05) |
| > 25 | 1,510 (4.5) | 63 | 0.96 (0.73, 1.25) |
| Note: Adjusted for maternal age, maternal education, maternal occupation, population density. Model was fitted using robust standard error to control for nonindependence of pregnancies by the same woman. NO_3_⁻, nitrate concentration in drinking water; CI, confidence interval; Ref, reference. | | | |
|  |  |  |  |

| **Table S6.** Cox regression for the association between pregnancy loss until gestational week 28 and drinking water nitrate including risk factors and confounders. | | | |
| --- | --- | --- | --- |
|  |  |  |  |
|  | n=93,051 (4,377 pregnancy loss) | | |
| NO_3_⁻ (mg/L) | n (%) | Pregnancy loss, (n) | aHR (95% CI) |
| Categorical |  |  |  |
| ≤ 1 | 27,655 (29.7) | 1,266 | Ref (1) |
| >1- ≤ 2 | 25,978 (27.9) | 1,244 | 0.97 (0.89, 1.05) |
| > 2- ≤ 5 | 24,689 (26.5) | 1,189 | 0.99 (0.91, 1.07) |
| > 5- ≤ 25 | 10,667 (11.5) | 490 | 0.97 (0.87, 1.07) |
| > 25 | 4,062 (4.4) | 188 | 0.95 (0.81, 1.10) |
| Note: Adjusted for maternal age, maternal education, maternal occupation, population density. Model was fitted using robust standard error to control for nonindependence of pregnancies by the same woman. NO_3_⁻, nitrate concentration in drinking water; CI, confidence interval; Ref, reference. | | | |
|  |  |  |  |
|  |  |  |  |
|  |  |  |  |

| **Table S7** Cox regression for the association between pregnancy loss until gestational week 22 and drinking water nitrate, stratified by maternal age at conception. Including risk factors and confounders. | | | | | | | | | | |
| --- | --- | --- | --- | --- | --- | --- | --- | --- | --- | --- |
|  |  |  |  |  |  |  |  |  |  |  |
|  |  |  |  |  |  |  |  |  |  |  |
|  |  | n=91,716 (4,212 pregnancy loss) | | | | | | | | |
|  |  | ≤ 25 years | | |  | > 25 years | | |  |  |
| NO3⁻ (mg/L) |  | Pregnancies (n(%)) | Pregnancy losses (n) | aHR (95% CI) |  | Pregnancies (n(%)) | Pregnancy losses (n) | aHR (95% CI) |  | *p* |
| ≤ 1 |  | 3,375 (31.2%) | 135 | Ref |  | 23,630 (29.6%) | 1,080 | Ref |  | 0.441 |
| >1- ≤ 2 |  | 2,959 (24.7%) | 122 | 1.10 (0.85, 1.40) | | 22,737 (28.5%) | 1,084 | 0.98 (0.90, 1.08) | |  |
| > 2- ≤ 5 |  | 3,113 (26.0%) | 134 | 1.17 (0.92, 1.49) | | 21,104 (26.5%) | 1,010 | 1.01 (0.93, 1.11) | |  |
| > 5- ≤ 25 |  | 1,568 (13.1%) | 60 | 1.01 (0.75, 1.37) | | 8,860 (11.1%) | 408 | 0.97 (0.87, 1.09) | |  |
| > 25 |  | 615 (5.1%) | 31 | 1.29 (0.89, 1.89) | | 3,395 (4.3%) | 148 | 0.88 (0.74, 1.05) | |  |
| Note: Adjusted for maternal education, maternal occupation, population density, BMI, Smoking and alcohol. Model was fitted using robust standard error to control for nonindependence of pregnancies by the same woman. NO3⁻, nitrate concentration in drinking water; CI, confidence interval; Ref, reference. | | | | | | | | | | |
|  |  |  |  |  |  |  |  |  |  |  |
|  |  |  |  |  |  |  |  |  |  |  |

**Table S8.** Adjusted hazard ratios (aHR) and 95% confidence intervals (CI's) of pregnancy loss associated with drinking water nitrate

exposure in pregnancy ~~Cox regression for the association between pregnancy loss until gestational week 22 and drinking water nitrate~~, stratified by nitrosatable drug use in pregnancy. ~~Including risk factors and confounders.~~

|  |  |  |  |  |  |  |  |  |  |  | | |
| --- | --- | --- | --- | --- | --- | --- | --- | --- | --- | --- | --- | --- |
|  |  | Nitrosatable drug exposure n=88,111 observations corresponding to 83,575 pregnancies (2,915 pregnancy losses)^a^ | | | | | | | | | | |
|  |  | No | | |  | Yes | | |  |  | | |
| NO_3_⁻ (mg/L) |  | Observations (n (%)) | Pregnancy losses (n) | aHR (95% CI) |  | Observations (n(%)) | Pregnancy losses (n) | aHR (95% CI) |  | *p* | | |
| ≤ 1 |  | 22,320 (29.6) | 774 | Ref (1) |  | 3,883 (30.3) | 57 | Ref (1) |  | 0.670 | | |
| >1- ≤ 2 |  | 21,338 (28.3) | 781 | 0.95 (0.86, 1.05) |  | 3,394 (26.5) | 48 | 0.89 (0.60, 1.30) | | |  |  |
| > 2- ≤ 5 |  | 19,812 (26.3) | 719 | 0.98 (0.88, 1.09) |  | 3,405 (26.6) | 62 | 1.22 (0.85, 1.74) |  |  | | |
| > 5- ≤ 25 |  | 8,561 (11.4) | 322 | 1.05 (0.92, 1.20) |  | 1,532 (12.0) | 24 | 1.07 (0.66, 1.73) |  |  | | |
| > 25 |  | 3,278 (4.4) | 117 | 1.00 (0.82, 1.21) |  | 588 (4.6) | 11 | 1.11 (0.58, 2.11) |  |  | | |
| Note: Adjusted for maternal age, maternal education, maternal occupation, population density, BMI, Smoking, alcohol and parity. Model was fitted using robust standard error to control for nonindependence of pregnancies by the same woman. NO_3_⁻, nitrate concentration in drinking water; CI, confidence interval; Ref, reference.  ^a^LMP to GA 21 weeks plus 6 days. | | | | | | | | | | | | |
